# The grounded psychometric development and initial validation of the Health Literacy Questionnaire (HLQ)

**DOI:** 10.1186/1471-2458-13-658

**Published:** 2013-07-16

**Authors:** Richard H Osborne, Roy W Batterham, Gerald R Elsworth, Melanie Hawkins, Rachelle Buchbinder

**Affiliations:** 1 Public Health Innovation, Population Health Strategic Research Centre, School of Health and Social Development, Deakin University, 221 Burwood Highway, Melbourne, Victoria 3125, Australia; 2 Monash Department of Clinical Epidemiology, Cabrini Hospital, Brighton, Victoria, Australia

**Keywords:** Health literacy, Measurement, Assessment, Health competencies, Psychometrics, HLQ

## Abstract

**Background:**

Health literacy has become an increasingly important concept in public health. We sought to develop a comprehensive measure of health literacy capable of diagnosing health literacy needs across individuals and organisations by utilizing perspectives from the general population, patients, practitioners and policymakers.

**Methods:**

Using a validity-driven approach we undertook grounded consultations (workshops and interviews) to identify broad conceptually distinct domains. Questionnaire items were developed directly from the consultation data following a strict process aiming to capture the full range of experiences of people currently engaged in healthcare through to people in the general population. Psychometric analyses included confirmatory factor analysis (CFA) and item response theory. Cognitive interviews were used to ensure questions were understood as intended. Items were initially tested in a calibration sample from community health, home care and hospital settings (N=634) and then in a replication sample (N=405) comprising recent emergency department attendees.

**Results:**

Initially 91 items were generated across 6 scales with agree/disagree response options and 5 scales with difficulty in undertaking tasks response options. Cognitive testing revealed that most items were well understood and only some minor re-wording was required. Psychometric testing of the calibration sample identified 34 poorly performing or conceptually redundant items and they were removed resulting in 10 scales. These were then tested in a replication sample and refined to yield 9 final scales comprising 44 items. A 9-factor CFA model was fitted to these items with no cross-loadings or correlated residuals allowed. Given the very restricted nature of the model, the fit was quite satisfactory: ***χ***^2^_WLSMV_(866 d.f.) = 2927, p<0.000, CFI = 0.936, TLI = 0.930, RMSEA = 0.076, and WRMR = 1.698. Final scales included: Feeling understood and supported by healthcare providers; Having sufficient information to manage my health; Actively managing my health; Social support for health; Appraisal of health information; Ability to actively engage with healthcare providers; Navigating the healthcare system; Ability to find good health information; and Understand health information well enough to know what to do.

**Conclusions:**

The HLQ covers 9 conceptually distinct areas of health literacy to assess the needs and challenges of a wide range of people and organisations. Given the validity-driven approach, the HLQ is likely to be useful in surveys, intervention evaluation, and studies of the needs and capabilities of individuals.

## Background

The World Health Organisation (WHO) describes health literacy as “the cognitive and social skills which determine the motivation and ability of individuals to gain access to, understand and use information in ways which promote and maintain good health”[1]. In both developing and developed countries, health and social policies are being developed that highlight health literacy as a key determinant of a person’s ability to optimally manage their health and of a health system’s ability to ensure equitable access to, and use of, services [2-4].

Low health literacy has been reported to be associated with increased mortality [5,6], hospitalisation [7,8], lower use of preventive healthcare services [9], poor adherence to prescribed medications [10], difficulty communicating with health professionals [11], and poorer knowledge about disease processes and self-management skills among people with chronic conditions such as diabetes, heart disease and arthritis [12-14]. Poor health literacy has also been linked with increased healthcare costs. A 1999 report by the USA National Academy on an Aging Society concluded that low health literacy increased national annual healthcare expenditures by $73 billion [15]. Studies also suggest that differences in health literacy abilities may explain observed health inequalities among people of different race, and with different educational attainments [16-18].

However, most of these studies used measures of health literacy that fail to capture the full breadth of ideas embodied in definitions of health literacy and they have also been shown to have substantive psychometric weaknesses [19,20]. The most widely used of these measures include the Rapid Estimate of Adult Literacy in Medicine, which tests reading ability and pronunciation [21]; the Test of Functional Health Literacy in Adults, which tests reading comprehension and numeracy [22]; and the Newest Vital Sign, which is a short clinical screening tool that assesses reading comprehension and numeracy using an ice cream label [23]. These measures return very different conclusions when applied concurrently [24,25]. At the population level, proxy measures such as the Adult Literacy and Life Skills Survey have been derived from national literacy surveys [19], but items and scoring are not publicly available and, like the individual measures, the categories poorly discriminate and provide little insight into actions that need to be taken to improve health literacy [19].

To address these shortcomings, we developed a comprehensive model of health literacy based upon concept mapping workshops and patient interviews [26] to derive the Health Literacy Management Scale (HeLMS) [26]. This tool has been used in several published [27] and unpublished studies, however experience with its use has led to the identification of some limitations. While it appears to be sensitive to serious health literacy limitations, it may be unable to detect less severe limitations. In addition, a scale within the HeLMS related to economic barriers to care, which was found to have the greatest variance, may be better considered a contextual, rather than primary, health literacy scale.

We subsequently conducted workshops at an international conference that focuses on outcomes measurement in rheumatology (OMERACT) where structured consultation with experts and patients led to the development of 98 statements and the identification of 16 major content domains for health literacy [28]. In comparing the HeLMs [26] with our initial concept mapping data (unpublished) and with data derived from the OMERACT workshop [28], we have found that the HeLMS covers less than half of the concepts from our initial consultations. This may be due to the fact that we excluded items on the basis of a modifiability criterion (the item had to be something that was potentially modifiable). Upon reflection, this criterion had been applied somewhat arbitrarily (e.g., it had not been applied to income issues). We had also neglected to consider that the measurement of health literacy is as much about identifying ways for services to accommodate people with different health literacy needs as it is about identifying ways of improving an individual’s health literacy.

Based upon these issues, we reanalysed the initial consultation data and the OMERACT data to develop a new model of health literacy from which we derived a new multidimensional health literacy profile, the Health Literacy Questionnaire (HLQ). This paper describes the conceptualisation, psychometric development and initial validation of the new tool. We sought to develop a tool that was capable of detecting a wide range of health literacy needs of people in the community, and that could be used for a variety of purposes from describing the health literacy of the population in health surveys through to measuring outcomes of public health and clinical interventions designed to improve health literacy.

## Methods

**Figure 1:**
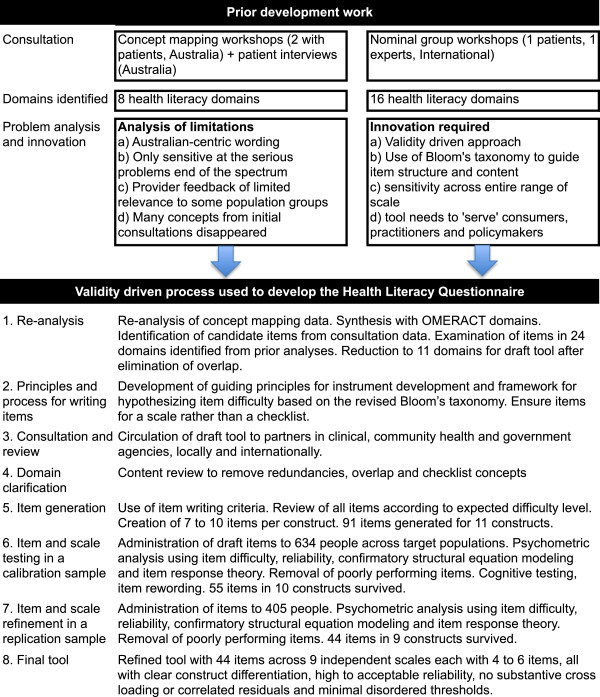
Steps undertaken in the development of the Health Literacy Questionnaire.

Figure 1 outlines the development of the new tool. We used a “validity-driven” instrument development approach [29] with structured processes governing the movement from consultation data to measurement tool. This initially involved the development of a comprehensive list of distinct concepts from the data and then developing a set of domains (guided by the way in which the concepts were grouped in the data) to accommodate all of these concepts (steps 1 to 3 in Figure 1). The second phase involved eliminating unimportant concepts and combining overlapping concepts to minimise the number of domains necessary to accommodate the remaining concepts. This step had a qualitative beginning and then proceeded with two psychometric steps (steps 5 to 7 Figure 1). The methods and outcomes from the prior concept mapping groups and patient interviews, and the OMERACT workshops, are described elsewhere [26,28]. This study was approved by the Deakin University and Barwon Health Human Research Ethics Committees.

### Reanalysis of concept mapping data and synthesis with OMERACT domains

The first task in the development of the HLQ was to reanalyse the concept mapping data that had been generated during the development of the HeLMs and to synthesise it with the nominal group data collected at OMERACT. The original concept maps were generated based upon a structured concept mapping process and software developed by Trochim [30-32]. In brief, the first step in concept mapping is a nominal group technique with a highly structured brainstorming process designed to maximise the breadth of ideas generated and equality of input from participants. This first brainstorming step involved participants responding to the following seeding statement; “Thinking broadly about your experiences in trying to look after your health, what abilities does a person need to have in order to get, understand, and use health information to make informed decisions about their health?” In this process, the ideas are printed onto cards, which are then sorted by individual participants in any way that makes sense to them. These sorts are combined using multi-dimensional scaling to produce a two-dimensional map in which statements that were sorted together by many participants sit close together, and statements that were rarely or never sorted together are far apart. The software then performs cluster analysis on the data and draws boundaries around groups of closely located items. The map is then discussed with participants who can refine the clusters by re-assigning items and who also name the clusters.

It is possible, however, to consider fewer or more cluster solutions depending upon whether the purpose is for more general or more precise descriptions, respectively. For example a three cluster solution may give the general insight that health literacy has major components including obtaining, evaluating and acting on information, whereas a fifteen cluster solution would provide a lot of detail about the elements that make up each of these major components. A tree diagram is a type of output from cluster analysis software that enables researchers to consider the substantive meaning of each split in a cluster when the number of clusters is increased. The maximum number of meaningful clusters represents the maximum number of distinct concepts that can be seen in the data. For instrument development this is critical in order to ensure that the construct being measured is fully represented.

To ensure that the maximum number of distinct concepts was identified from the original concept mapping and nominal group data, we used a tree diagram to examine increasingly precise breakdowns of groups of items. The ways in which these were grouped in each data set were then examined and an hypothesised set of domains, that was sufficient to cover all of the identified concepts, was developed. We constantly checked the statements and emerging domains of the new tool against the initial consultation data to ensure that salient concepts were being retained.

### Principles and processes for writing questionnaire items

#### Item difficulty based upon the revised Bloom’s taxonomy

One of the main priorities in developing the HLQ was to ensure that items in each scale collectively covered the full spectrum of health literacy capability so that the eventual scales would be sensitive for people with mild, moderate or severe health literacy limitations. We sought to ensure that the scales were capable of detecting small changes at all levels of health literacy capacity. To this end we constructed scales that had items with a range of ‘difficulty’ levels such that a more difficult item is one for which fewer people would give a maximum score. We used the revised Bloom’s taxonomy to guide the writing of items with higher difficulty [33-36]. This taxonomy organises cognitive tasks on two dimensions, each of which involves increasing complexity. The first dimension describes levels of knowledge including factual, cognitive, procedural and meta-cognitive knowledge, while the second dimension describes increasingly demanding cognitive processes including remembering, understanding, applying, analysing, evaluating and creating. The two dimensions are not independent and they interact. In general, higher levels require at least some level of attainment at each of the lower levels. In this sense the cognitive tasks at higher levels are more difficult in that they require attainment of the lower level skills and then some additional level of knowledge or skill. For example, in asking respondents to indicate their level of agreement with the statement ‘I have a health professional that I trust to help me make decisions about my health’ adds the concept of decision-making to the statement ‘I have at least one health professional that I trust’. Higher-order items are less likely to achieve maximum ratings and would thus increase the range of health literacy needs that the scale could detect.

#### Nature of item content

An additional consideration was whether items in a domain were likely to form a scale or whether they should be treated as a checklist of contextual factors. The two areas where we had to make this decision related to health beliefs and barriers to access to health services. While conceptually related, factors affecting access to services such as affordability, proximity and cultural appropriateness could not be added together to give a scale score and when this was attempted all factors other than economic factors had to be deleted on psychometric grounds. The same was true of various health beliefs highlighted in the consultation data. Health beliefs such as that poor health is inevitable with old age, or that health is just a matter of luck or fate, or that vaccinations are dangerous, could not be additively combined and are best treated as a checklist.

#### Item generation

The statements within each candidate domain of health literacy informed the development of items. Items were refined to generate increasingly cogent constructs. This process was facilitated by specifying, within each construct, two vignettes – one of an individual with very high levels of the construct’s attributes and one with very low levels of the attributes. This facilitated the development of items that covered the full range of respondents’ potential extant health literacy attributes. At all times, direct quotes and words used by the workshop participants were used to maximise content and face validity.

To ensure balanced coverage of identified concepts within draft constructs, statements within hypothesised scales were reviewed to identify any potential sub-themes. One to three within construct sub-themes were identified and were used as the basis to generate an even number of items within each subgroup. There was constant referral back to the high/low vignettes and, iteratively, the items and the construct definitions were refined as both items and constructs became more clearly defined. We sought to write about 50% more items than what would be required in the final scale with a minimum of 4 items per scale set a priori.

Item generation was highly structured with constant reference to how a wide range of respondents might attend to each item. Each item was required to be succinct with only one or two cognitive decisions required for an answer to be generated by a respondent. It was considered that the delivery format was to be varied (oral, paper or computer formats) given that respondents may have low literacy, might be ill or may have English as a second language. The content had to be immediately relevant to respondents with a range of experiences: in a state of good or poor health; in receipt of healthcare from medical through to health promotion activities; with extensive or minimal experience of health and social systems; and across the age, sex, education, and cultural spectrums.

Once a set of items was drafted, Bloom’s taxonomy was used as a guide to further refine the items to ensure a wide range of difficulty was embedded within each scale. The items were primarily generated by three of the authors (RHO, RB, RWB) who have extensive experience of working with individuals from across a wide range of communities, and of writing items for questionnaire development. Response options for each domain were determined by the content and nature of the generated items.

#### Consultation and review

The items and the constructs were emailed to several groups for informal review: clinical staff at four community health centres in Melbourne who serve catchments with low and high socioeconomic status; staff at the Victorian Department of Health who administer programs for home services, community nursing and primary care; a researcher with experience in indigenous health and development; and a qualitative researcher with medical editing expertise. The draft was also presented, opportunistically, to experts and colleagues in a range of other countries and cultures. The purpose of these reviews was for experts from a wide range of backgrounds to provide feedback on the relevance and appropriateness of the items and concepts to their settings.

### Statistical analyses

Descriptive statistics were generated for each item to determine the extent of missing values and floor and ceiling effects across domains. Given that hypothesised constructs were specified a priori, confirmatory factor analysis (CFA) was used. Items were firstly administered to a calibration sample and the number of items reduced according to the protocol below. The refined set of items was then re-administered to a replication sample to verify the items and scales.

Item selection and scale validation were conducted in the tradition of classical test theory using: a) recent programming for point and interval estimation of item difficulty, item-remainder correlations and composite reliability if an item is removed; b) restricted factor analysis (often termed confirmatory factor analysis – CFA); and c) exploratory structural equation modeling (ESEM) procedures. All analyses were conducted with Mplus Version 6, which provides full information maximum-likelihood estimation for missing data for the analysis of ordinal variables that uses all available data on all items.

The first analyses provided estimates of item “difficulty”, item-remainder correlations and scale reliability if the item were to be deleted. While these analyses mirrored the classical item analysis procedures available in general statistical analysis programs the item analysis routines in these programs provide only point estimates of sample values and are typically based on the calculation of Pearson product–moment correlation coefficients, which are inappropriate for the analysis of four and five-point ordinal response options used in the HLQ. Recently Raykov [37] has published the statistical theory and code for structural equation modeling programs (e.g., MPlus) for the calculation of these classical scale evaluation statistics based on the polychoric and polyserial correlations appropriate for the analysis of ordinal data. Further, it is well known that Cronbach’s coefficient alpha, a widely-used index of composite scale reliability, is frequently a biased estimate of population reliability, both in cases where the scale components are not tau-equivalent (i.e., do not have equal factor loadings) [38] and where item errors may be correlated [37]. Both conditions are likely to apply in the HLQ. Raykov’s procedures provide unbiased estimates of composite reliability that avoid these limitations of Cronbach’s alpha. A further advantage is that these procedures link directly to the CFA of the hypothesised scales and thus provide a coherent program of item analysis and collection of statistical evidence for scale consistency (unidimensionality) and reliability and, when extended to multi-scale analyses, discriminant validity.

For scales with agree/disagree response options, the “difficulty” level was calculated as the proportion responding Disagree and Strongly disagree as against Agree or Strongly agree. For the competency scales, difficulty was calculated as the proportion responding Cannot do, Very difficult, or Quite difficult as against Quite easy and Very easy.

Following the classical item analysis, a one-factor CFA model was fitted to the data for each proposed scale. The focus here was to locate a model that yields a set of items that has maximum internal consistency (unidimensionality), other things being equal (e.g., criteria associated with item difficulty). Internal consistency/unidimensionality is defined as the model having acceptable fit to the data [39].

Consistency/unidimensionality of individual scales in multi-scale inventories is particularly important when these inventories are to be used for needs assessment and/or program evaluation where unambiguous construct measurement is essential. This was achieved using estimates of item-remainder correlations and, other things being equal, deleting items with the lowest estimates, sequential fitting of one-factor CFA models, and assessment of model fit.

CFA models were fitted to the data using the weighted least squares mean and variance adjusted (WSLMV) estimator available in MPlus. It is a diagonally-weighted least squares approach where only the diagonal elements of the weight matrix are used in the estimation while the full weight matrix is used to compute standard errors and ***χ***^2^[40].

Unstandardised and standardised factor loadings, an estimate of the variance in the measured variable explained by the latent variable (R^2^), and associated standard errors are provided in Mplus 6 together with fit statistics (***χ***^2^, CFI – Comparative Fit Index, TLI – Tucker-Lewis Index, RMSEA – Root Mean Square Error of Approximation, and WRMR – Weighted Root Mean Square Residual). Indicative threshold values for the tests of ‘close fit’ used in this analysis were CFI>0.95; TLI>0.95; RMSEA<0.06 and WRMR<1.0) while a value of <0.08 for the RMSEA was taken to indicate a ‘reasonable’ fit [41-43]. Mplus also provides statistics that can be used to facilitate model improvement by suggesting fixed parameters (e.g., in the case of single-factor models, correlations among residual variances) that might be freely estimated. In Mplus, these statistics include standardised residuals, modification indices (MIs) and the associated change in a parameter if the modification is included in the model (Standardised Expected Parameter Change – SEPC).

MPlus also provides a method of Item Response Theory (IRT) analysis that fits a polytomous IRT model to ordinal data using Samejima’s graded response model. An IRT analysis of the full set of items in each hypothesised scale was used as a complement to the ordinal CFA (WLSMV) model to check item thresholds. The thresholds of ordinal items (the point at which it is equally likely that a person will be classified into adjacent categories on the ordered responses) when arranged along the continuum of the latent variable should follow the response order (Strongly disagree, Disagree etc.). If this does not occur the thresholds are said to be disordered. Items with disordered thresholds do not classify respondents in the same order as the underlying continuum, an argument for not including the item in a summated scale.

Following the one-factor models, analyses of discriminant validity were conducted utilizing a series of multi-factor models; initially the scales with 4-point disagree/agree response options and 5-point cannot do/very easy response options were analyzed separately, while a final 9-factor CFA analysis was also conducted. These analyses focused on model fit and MIs and SEPCs that suggested that cross-loadings should be included in the model. Indications for improvement were followed up by including the relevant cross-loading in the model and obtaining estimates of its size (with confidence intervals) compared with the originally hypothesised loading.

Given the tendency for multi-scale CFA models with all potential cross-loadings fixed precisely to 0.0 to provide inflated estimates of inter-factor correlations [44], correlations of up to 0.95 are regarded as acceptable and may suggest the presence of a higher-order factor.

### Cognitive testing

This procedure involved initial administration of items using paper and pen format with careful observation of each respondent. The interviewer then went back through items with the respondent and specifically probed them on items if they had hesitated or appeared to have found an item difficult to answer. They were asked “What were you thinking about when you were answering that question?”. This process elicited the cognitive process behind their answers. A prompt question was used if needed: “Why did you select that answer?”.

## Results

### Domain clarification and specification of hypothesised constructs

Statements and constructs arising from concept mapping (2 workshops, each giving rise to 9 constructs and a total of 81 statements) and nominal group workshops (16 constructs with 98 statements) were synthesised to generate 13 separate initial constructs (extreme left of Table 1.). Content analysis suggested that these constructs were spread across three overarching areas: About self, Dealing with the outside world, and Being resourced.

**Table 1:** Evolution of the constructs and scales to define health literacy

**Initial construct descriptor**	**Overarching theme**	Draft scales tested in calibration sample*	Refined scales tested in replication sample**	Final scale **	**Response options**
1. Characteristics of relationships with providers (regular, trust)	1 About self	1 Healthcare provider support [9]	1 Healthcare provider support [6]	1. Feeling understood and supported by healthcare providers [4]	Agree/ disagree
2. Dealing with providers (2^nd^ opinion/ assertiveness/ communication/ questions/ listens), difference between new and familiar providers	2 Dealing with outside world	2 Agency in relationships with providers [8]	2 Agency in relationships with healthcare providers [5]	6. Ability to actively engage with healthcare providers [5]	Difficulty
3. Skills of using health system	2 Dealing with outside world	3 Skills for using the health system [6]	3 Navigating the health system [6]	7. Navigating the health system [6]	Agree/ disagree
4. Practicalities of accessing health system	2 Dealing with outside world	4 Practicalities of accessing the health system [9]	[Re-classified as checklist assessment]		Difficulty
5. Cognitive barriers/strategies. memory; organisation; judgement; not getting overwhelmed and confused	1 About self	[Subsumed into Critical appraisal]			
6. Understanding and critical appraisal	3 Being resourced	5 Critical appraisal [7]	4 Critical appraisal [6]	5. Appraisal of health information [5]	Agree/ disagree
7. Skills/options for accessing information	1 About self	6 Ability to access information [10]	5 Ability to access information [5]	8. Ability to find good quality health information [5]	Difficulty
8. Having condition related information, information and knowledge adequacy	3 Being resourced	7 Perceived information adequacy [7]	6 Perceived information adequacy [7]	2. Having sufficient information [4]	Difficulty
9. Self management/ motivation/ prioritisation	2 Dealing with outside world	8 Taking responsibility for health [10]	7 Taking responsibility for health [5]	3. Actively managing my health [5]	Difficulty
10. Being health focused. Where your head is (psych/ emotional state/ self discipline/ will power/ acceptance of condition)	1 About self	9 Health focused [9]	8 Health focused [6]	Engagement in decisions. [Scale removed due to items not having sufficient difficulty range]	
11. Social support	3 Being resourced	10 Social support [7]	9 Social support [5]	4. Social support for health [5]	Agree/ disagree
12. Literacy and numeracy	2 Dealing with outside world	11 Understanding health and healthcare [8]	10 Reading and writing health information [5]	9. Understand health information well enough to know what to do [5]	Agree/ disagree
13. Beliefs and values	1 About self	[Inventory style assessment]			

* values in parenthesis indicates the number of items in the scale.

** number of surviving items in the scale.

Inspection of the statement content revealed that Cognitive barriers had substantial overlap with Understanding and critical appraisal and these were combined to form a construct labelled Critical appraisal. Analysis of the Beliefs and values domain indicated that it was an incomplete checklist of contextual factors and was excluded. Similarly, the Practicalities of accessing the health system dimension was not included because the concepts were found to focus on the context of a person’s life, including physical accessibility and barriers related to health literacy. A person could have excellent health literacy across a range of dimensions but they may have physical and environmental challenges that prevented them from accessing health services.

Analysis of the content of the statements within draft constructs enabled development of vignettes of individuals with low and high levels of the construct’s implied attributes (see Table 2). Constant checking of item content across all draft constructs, and the item generation process (see next section) produced increasing clarity of the construct names, vignettes, and individual items.

**Table 2:** The Health Literacy Questionnaire scales with high and low descriptors of each construct

**Low level of the construct**	**High level of the construct**
**1. Feeling understood and supported by healthcare providers**	
People who are low on this domain are unable to engage with doctors and other healthcare providers. They don’t have a regular healthcare provider and/or have difficulty trusting healthcare providers as a source of information and/or advice.	Has an established relationship with at least one healthcare provider who knows them well and who they trust to provide useful advice and information and to assist them to understand information and make decisions about their health.
**2. Having sufficient information to manage my health**	
Feels that there are many gaps in their knowledge and that they don't have the information they need to live with and manage their health concerns.	Feels confident that they have all the information that they need to live with and manage their condition and to make decisions.
**3. Actively managing my health**	
People with low levels don’t see their health as their responsibility, they are not engaged in their healthcare and regard healthcare as something that is done to them.	Recognise the importance and are able to take responsibility for their own health. They proactively engage in their own care and make their own decisions about their health. They make health a priority.
**4. Social support for health**	
Completely alone and unsupported for health.	A person’s social system provides them with all the support they want or need for health.
**5. Appraisal of health information**	
No matter how hard they try, they cannot understand most health information and get confused when there is conflicting information.	Able to identify good information and reliable sources of information. They can resolve conflicting information by themselves or with help from others.
**6. Ability to actively engage with healthcare providers**	
Are passive in their approach to healthcare, inactive i.e., they do not proactively seek or clarify information and advice and/or service options. They accept information without question. Unable to ask questions to get information or to clarify what they do not understand. They accept what is offered without seeking to ensure that it meets their needs. Feel unable to share concerns. The do not have a sense of agency in interactions with providers.	Is proactive about their health and feels in control in relationships with healthcare providers. Is able to seek advice from additional healthcare providers when necessary. They keep going until they get what they want. Empowered.
**7. Navigating the healthcare system**	
Unable to advocate on their own behalf and unable to find someone who can help them use the healthcare system to address their health needs. Do not look beyond obvious resources and have a limited understanding of what is available and what they are entitled to.	Able to find out about services and supports so they get all their needs met. Able to advocate on their own behalf at the system and service level.
**8. Ability to find good health information**	
Cannot access health information when required. Is dependent on others to offer information.	Is an 'information explorer'. Actively uses a diverse range of sources to find information and is up to date.
**9. Understanding health information well enough to know what to do**	
Has problems understanding any written health information or instructions about treatments or medications. Unable to read or write well enough to complete medical forms.	Is able to understand all written information (including numerical information) in relation to their health and able to write appropriately on forms where required.

### Item generation

The number of items per domain that were submitted for testing in the field is shown in Table 1 and ranged from 7 to 10. The concepts in items that represented personal attributes, resources or approaches fitted well with an disagree/agree Likert scale, whereas the remaining scales pertained to specific or general competences and fitted better with a cannot do to very easy scale.

### Item and scale testing in a calibration sample

The 91 items were administered to 634 people from target settings including people attending a private specialist rheumatology clinic at Cabrini Health community hospital (n=63); metropolitan organisations providing Home and Community Care (n=411); and people who had attended the emergency department at Barwon Health (n=160), a large regional teaching hospital, between 2 and 6 months earlier. The mean (SD) age was 65 (19) years, 69% were female, 55% had a high school education or less, and 19% required assistance to complete the form due to insufficient English language, sight or other impairment (see Table 3). Table 1 shows the number of starting and number of surviving items. Only a summary of the extensive calibration dataset analysis is presented here.

**Table 3:** Demographic profile of participants in calibration and replication samples

	Sample*			
	**Calibration**		**Replication**	
Setting				
Barwon Health Emergency Department	160	25%	405	100%
Cabrini Health community hospital	63	10%		
Home and Community Care	411	65%		
Age (mean, SD)	64.7	19.1	49.2	19.7
Female	409	69%	246	61%
Assisted to complete form	111	19%	28	7%
Education				
Primary school or less	51	8%	8	2%
High school (not completed)	160	25%	98	24%
High school (completed)	137	22%	71	18%
TAFE/Trade	191	30%	102	25%
University	45	7%	122	30%
Private health insurance	316	55%	230	56%
Receive Government benefit	391	68%	185	45%
Live alone	187	33%	68	17%
Long-standing illness or disability (more than one possible)				
Musculoskeletal (arthritis, osteoporosis, back pain or other)	305	52%	212	52%
Depression, anxiety or other mental health condition	118	20%	87	22%
Heart disease	102	18%	43	11%
Diabetes	91	16%	29	7%
Asthma, emphysema or other respiratory condition	78	13%	73	18%
Cancer	38	7%	25	6%
Stroke, multiple sclerosis or other neurological condition	39	7%	16	4%

* Number and percentage unless otherwise stated.

Among the scales applied in the calibration sample, the scale with the widest range of difficulty scores for items was Ability to access health information: 59% of respondents scored in the difficult categories for the hardest item down to 22% providing these responses for the easiest item. This scale also had the ‘hardest’ items overall (median difficulty score = 0.36). The scale with the easiest items was Feeling understood and supported by healthcare providers with a median difficulty of 0.10 and the proportion of difficult items ranging from 6-16%.

The composite reliability for all unrefined scales was acceptable at this stage of their development – the lowest reliability estimate being 0.77 for Critical appraisal. For most of the scales, the fit of a one-factor confirmatory factor analysis model was not satisfactory, but was considerably improved as poorly performing items were eliminated. Four items among the cannot do/very easy scales were found to have disordered thresholds. As outlined above, multi-factor measurement models were initially fitted to the full set of items for the disagree/agree scales and separately for the cannot do/very easy scales with the aim of maximising item homogeneity through elimination of items exhibiting large cross-loadings or inter-factor correlated residuals. One item from the Critical appraisal scale exhibited some cross-loading (positive cross-loadings from Social support and Actively managing my health) was retained but “tagged” for possible removal at a later stage. Items were thus removed mainly on the basis of the one-factor modeling (factor loadings clearly lower than those of other items in the scale, unacceptably high intra-factor correlated residuals, clearly higher composite reliability if item deleted, and disordered thresholds).

Overall, one-factor models for the final selection of items fitted the data reasonably well. According to the “close fit” criteria outlined above, one scale showed good fit to the data across all indices and one acceptable fit (RMSEA ≥0.06 but <0.08). The model fit for seven scales was not acceptable on the basis of a RMSEA ≥0.08. However, for all models the CFI, TLI and WRMR were within the pre-specified cut-off criteria. As the RMSEA tends to show better fit than the CFI and TLI in models with large numbers of variables [45] and, conversely, worse fit with models with a small number of variables, and as model fit was clearly acceptable on the other three indices specified we believe that all models demonstrated an acceptable level of construct homogeneity. The items retained for each scale had acceptable loadings on their respective factors with the exception of two items in the Critical appraisal scale (loadings of 0.54 and 0.56).

The 55 items retained across the 10 scales were then tested in 11 cognitive interviews as a final check before they were retested in a replication sample. Overall, all items were found to be clearly understood by respondents.

The main reason for hesitation or apparent difficulty with answering items was that the respondent was reflecting on their own situation before answering (e.g., which information they might have access to, or who they might ask for help if they needed it). Responses informed minor word changes (e.g., ‘I can get access to “several people” who understand and support me’ instead of …plenty of people…”), and a definition of “healthcare provider” was added to the front cover of the questionnaire.

### Item and scale refinement in a replication sample

The 55 retained items across 10 scales were posted to 3,000 people who had attended the emergency department at Barwon Health, a large regional teaching hospital, between 2 and 6 months earlier. We targeted younger people; 40% were 18 to 30 years old, 30% were 30 to 40 years old, and 30% were 40 years and older. Younger people are less likely to have chronic conditions or prior experience with the healthcare system. 412 (13.7%) people responded to the invitation pack which consisted of a letter of invitation to complete the health literacy questionnaire (entitled the “Understanding health and healthcare questionnaire”), the 55 items in questionnaire format, and a set of demographic questions. The mean (SD) age was 49.2 years, 61% were female and 44% had a high school education or less. Over 50% of respondents reported a musculoskeletal condition and 21.5% reported having depression, anxiety or other mental health condition. See Table 3 for a full description of the respondents.

The proportion of non-response to items was small and varied between 1.4 and 2.9% suggesting that items were well understood and had acceptable content.

Table 4 shows the final psychometric properties of items and scales. The items selected were the best available indicators of the intended construct as indicated by highest item-remainder correlations and highest standardised factor loadings in one-factor models. In each scale, the items form a homogeneous cluster as indicated by a satisfactory close fit of a one-factor model. We ensured that minimal intra-factor correlated residuals were present, particularly if specific content or linguistic overlap was evident. In-depth revision of the item content of the Critical appraisal scale revealed that it was better represented by the label ‘Appraisal of health information’ and was renamed. For all scales, a composite reliability of ≥0.8 was sought and achieved for all scales except Appraisal of health information (0.77) and Health Focus (0.78), however the median reliability was 0.88, with Ability to actively engage with healthcare providers with the highest (0.90).

**Table 4:** Psychometric properties of final HLQ items * and scales

		**Difficulty**	**Ordered**	**Factor Loading**	**R**^**2**^
		**(95% CI)**		**(95%CI)**	
**1. Feeling understood and supported by healthcare providers**					
1	I have at least one healthcare provider who…	0.19 (0.15-0.24)	Yes	0.84 (0.80- 0.87)	0.71
2	I have at least one healthcare provider I can…	0.10 (0.07-0.13)	Yes	0.99 (0.97- 1.01)	0.98
3	I have the healthcare providers I need…	0.18 (0.15-0.22)	Yes	0.77 (0.72- 0.81)	0.58
4	I can rely on at least one…	0.10 (0.08-0.13)	Yes	0.91 (0.87- 0.94)	0.82
	Model Fit – ***χ***^2^_WLSMV_(2) = 10.15, p= 0.0063, CFI = 0.998, TLI = 0.995, RMSEA = 0.100, and WRMR = 0.367.				
	Composite reliability = 0.88 (0.86-0.90)				
**2. Having sufficient information to manage my health**					
1	I feel I have good information about health…	0.11 (0.08-0.14)	Yes	0.73 (0.67-0.79)	0.54
2	I have enough information to help me deal…	0.21 (0.18-0.26)	Yes	0.88 (0.85-0.91)	0.77
3	I am sure I have all the information I…	0.27 (0.22-0.31)	Yes	0.98 (0.96-1.00)	0.96
4	I have all the information I need to	0.25 (0.21-0.30)	Yes	0.93 (0.91-0.95)	0.86
	Model Fit – ***χ***^2^_WLSMV_(2) = 5.24, p= 0.0730, CFI = 1.000, TLI = 0.999, RMSEA = 0.063, and WRMR = 0.337.				
	Composite reliability = 0.88 (0.87-0.90)				
**3. Actively managing my health**					
1	I spend quite a lot of time actively managing…	0.30 (0.25-0.34)	Yes	0.79 (0.74-0.84)	0.63
2	I make plans for what I need to do to be…	0.15 (0.12-0.18)	Yes	0.83 (0.79-0.87)	0.69
3	Despite other things in my life, I make time…	0.21 (0.17-0.25)	Yes	0.91 (0.87-0.95)	0.82
4	I set my own goals about health and fitness	0.13 (0.10-0.17)	Yes	0.72 (0.66-0.77)	0.52
5	There are things that I do regularly…	0.20 (0.16-0.24)	Yes	0.89 (0.85-0.92)	0.78
	Model Fit – ***χ***^2^_WLSMV_(5) = 31.96, p<0.0001, CFI = 0.992, TLI = 0.983, RMSEA = 0.115, and WRMR = 0.775.				
	Composite reliability = 0.86 (0.84-0.88)				
**4. Social Support for health**					
1	I can get access to several people who…	0.16 (0.13-0.20)	Yes	0.70 (0.64-0.75)	0.48
2	When I feel ill, the people around me really…	0.30 (0.26-0.35)	Yes	0.74 (0.69-0.80)	0.55
3	If I need help, I have plenty of people I…	0.18 (0.14-0.22)	Yes	0.88 (0.85-0.91)	0.77
4	I have at least one person…	0.19 (0.15-0.23)	Yes	0.72 (0.67-0.77)	0.52
5	I have strong support from…	0.10 (0.08-0.14)	Yes	0.89 (0.86-0.93)	0.79
	Model Fit – ***χ***^2^_WLSMV_(5) = 37.36, p<0.0001, CFI = 0.987, TLI = 0.975, RMSEA = 0.126, and WRMR = 0.925.				
	Composite reliability = 0.84 (0.81-0.86)				
**5. Appraisal of health information**					
1	I compare health information from different	0.18 (0.15-0.22)	Yes	0.68 (0.62- 0.74)	0.46
2	When I see new information about health, I…	0.38 (0.34-0.43)	Yes	0.73 (0.67- 0.79)	0.53
3	I always compare health information from…	0.34 (0.30-0.39)	Yes	0.86 (0.81-0.90)	0.74
4	I know how to find out if the health…	0.30 (0.25-0.34)	Yes	0.59 (0.51- 0.66)	0.34
5	I ask healthcare providers about the quality…	0.38 (0.33-0.43)	Yes	0.62 (0.55-0.68)	0.38
	Model Fit – ***χ***^2^_WLSMV_(5) = 18.05, p= 0.0029, CFI = 0.990, TLI = 0.980, RMSEA = 0.080, and WRMR = 0.610				
	Composite reliability = 0.77 (0.74-0.81)				
**6. Ability to actively engage with healthcare providers**					
1	Make sure that healthcare providers understand…	0.23 (0.19-0.27)	Yes	0.79 (0.75- 0.84)	0.63
2	Feel able to discuss your health concerns with a…	0.15 (0.11-0.18)	Yes	0.88 (0.85- 0.90)	0.77
3	Have good discussions about your health…	0.18 (0.14-0.22)	Yes	0.85 (0.82- 0.88)	0.72
4	Discuss things with healthcare providers…	0.23 (0.19-0.28)	Yes	0.87 (0.84-0.90)	0.76
5	Ask healthcare providers questions to get…	0.24 (0.20-0.28)	Yes	0.88 (0.85- 0.91)	0.77
	Model Fit – ***χ***^2^_WLSMV_(5) = 74.91, p<0.0001, CFI = 0.986, TLI = 0.973, RMSEA = 0.185, and WRMR = 0.944.				
	Composite reliability = 0.90 (0.88-0.92)				
**7. Navigating the healthcare system**					
1	Find the right healthcare	0.19 (0.16-0.23)	Yes	0.76 (0.71-0.80)	0.57
2	Get to see the healthcare providers I need to	0.07 (0.05-0.10)	No	0.61 (0.54-0.68)	0.37
3	Decide which healthcare provider you need…	0.20 (0.17-0.24)	Yes	0.92 (0.89-0.94)	0.84
4	Make sure you find the right place to get…	0.19 (0.16-0.23)	Yes	0.94 (0.92-0.96)	0.88
5	Find out what healthcare services you are…	0.42 (0.37-0.47)	Yes	0.77 (0.73-0.82)	0.60
	6 Work out what is the best care for you	0.28 (0.24-0.33)	Yes	0.80 (0.76-0.83)	0.63
	Model Fit – ***χ***^2^_WLSMV_(9) = 21.74, p= 0.0097, CFI = 0.998, TLI = 0.996, RMSEA = 0.058, and WRMR = 0.451.				
	Composite reliability = 0.88 (0.87-0.90)				
**8. Ability to find good health information**					
1	Find information about health problems	0.21 (0.17-0.25)	Yes	0.85 (0.81-0.88)	0.72
2	Find health information from several…	0.27 (0.23-0.32)	Yes	0.86 (0.83-0.89)	0.74
3	Get information about health so you are…	0.23 (0.19-0.27)	Yes	0.87 (0.84-0.89)	0.75
4	Get health information in words you…	0.20 (0.17-0.24)	Yes	0.81 (0.77-0.85)	0.66
5	Get health information by yourself	0.26 (0.22-0.30)	Yes	0.84 (0.80-0.87)	0.70
	Model Fit – ***χ***^2^_WLSMV_(5) = 57.06, p<0.0001, CFI = 0.989, TLI = 0.977, RMSEA = 0.160, and WRMR = 0.820.				
	Composite reliability = 0.89 (0.87-0.91)				
**9. Understanding health information well enough to know what to do**					
1	Confidently fill medical forms in the correct…	0.13 (0.10-0.17)	No	0.80 (0.75-0.84)	0.63
2	Accurately follow the instructions from…	0.08 (0.06-0.21)	No	0.82 (0.77-0.87)	0.67
3	Read and understand written health…	0.15 (0.12-0.19)	Yes	0.84 (0.81-0.88)	0.71
4	Read and understand all the information on…	0.16 (0.13-0.20)	Yes	0.83 (0.79-0.87)	0.69
5	Understand what healthcare providers are…	0.14 (0.11-0.17)	Yes	0.88 (0.85-0.92)	0.78
	Model Fit – ***χ***^2^_WLSMV_(5) = 35.70, p<0.0001, CFI = 0.992, TLI = 0.983, RMSEA = 0.123, and WRMR = 0.671				
	Composite reliability = 0.88 (0.86-0.90)				

* Items are truncated. Full items are available from the authors.

A 9-factor CFA model was fitted to the finally selected 44 items with no cross-loadings or correlated residuals allowed. Given the very restricted nature of the model, the fit was quite satisfactory: ***χ***^2^_WLSMV_(866 d.f.) = 2927.60, p<0.0000, CFI = 0.936, TLI = 0.930, RMSEA = 0.076, and WRMR = 1.698. While the CFI and TLI are lower than the pre-specified cut-off and the WRMR is higher, this is not surprising given the large number of parameters in the model set precisely to 0.0. The ranges of the factor loadings in this model (not shown) were: Feeling understood and supported by healthcare providers 0.79 – 0.95; Having sufficient information to manage my health 0.89 – 0.94; Actively managing my health 0.76 – 0.88; Social support for health 0.75 – 0.92; Appraisal of health information 0.54 – 0.92; Ability to actively engage with healthcare providers 0.81 – 0.91; Navigating the healthcare system 0.71 – 0.90; Ability to find good health information 0.81 – 0.89; and Understanding health information 0.76 - 0.93.

Correlations between factors showed a clear discrimination between the disagree/agree scales (range of inter-factor correlations, 0.43-0.78), however, clear discrimination was less evident for the scales within the cannot do/very easy scales (range of inter-factor correlations, 0.83-0.93) suggesting higher order factors may be present, including a general capability to interact positively and effectively with the healthcare system. While the Health focus scale mostly had acceptable properties (e.g., reasonable factor loadings and item total correlation, and good model fit with two minor negative correlated errors >0.2), it did have other weaknesses. First, there was some conceptual overlap with the Actively managing my health scale, and one of the items, ‘Despite other things in my life, I make time to be healthy’ fitted better in that scale and was consequently moved there. Further, for two items, the most extreme response option (strongly disagree) was never endorsed and all but one item (Despite other things in my life…) had low to very low difficulty. For the most difficult item, only 11% scored in the difficult categories, and for three items only 2% scored in the difficult categories. Given that the items were generally very easy and were unlikely to distinguish between people with different levels of health literacy, the scale was removed.

For the five scales with 4-point response options (strongly disagree to strongly agree) the scale with the smallest difficulty range was Feeling understood and supported by healthcare providers where the most difficult item was ‘I have at least one healthcare provider who knows me well’ (difficulty = 19%). The easiest two items (10%) included one pertaining to having at least one healthcare provider they could discuss health problems with, and the other being able to rely on at least one healthcare provider. The scale with the most difficult items was Appraisal of health information where four items had a difficulty of 30% or more. One of the hardest items was ‘I ask healthcare providers about the quality of the health information I find’ (38% response in the difficult categories). The easiest item in this scale was ‘I compare health information from different sources’ (18%). The other scales had items with item difficulty that ranged from 10% to 30%.

For scales 6 to 9 with a 5-point response continuum, the most difficult scale was Ability to find good health information with all five items having a difficulty of 20% or greater. The hardest item (Find out what healthcare services you are entitled to) with 42% of responses in the difficult categories was in the Navigating the health system scale. The content of this item reflected high Bloom’s taxonomy challenge. The easiest scale was Understand health information where the hardest item’s difficulty was 16% which reflected the lowest Bloom’s challenge, i.e., understanding.

To further improve the content validity and measurement precision, minor wording changes were undertaken in four items:

In the Social support scale, the word “people” in the item ‘I have people who can come to medical appointments with me’, was changed to “at least one person”. The term “people” was found to be ambiguous, the item had a correlated error with another item in the scale (I have strong support from family or friends), and the factor loading was somewhat lower (0.72).

In the Appraisal of health information scale, the words “for me” were removed from the item ‘I know how to find out if the health information I receive is right for me or not’. This item had the lowest factor loading (0.59), the scale had a relatively low reliability (0.77), and the deleted words appeared redundant and an unnecessary cognitive step.

In the Navigating the healthcare system scale, the item ‘Work out how to make an appointment to see a healthcare provider’ was simplified to ‘Get to see the healthcare providers I need to’. The idea of “working out” was regarded as slightly different to the notion of “navigating”, the item had the lowest factor loading (0.61), and the item had low difficulty (7%).

In the Understanding health information scale, the item ‘Follow the instructions from healthcare providers properly’ was changed to ‘Accurately follow the instructions from healthcare providers’ as the item had disordered thresholds, and the removal of the item reduced model fit and construct breadth.

Given that the wording of these items changed, albeit in minor ways, for the final version the specific item difficulty, together with the general and specific model parameters (fit, reliability, item thresholds, loadings etc.) associated with all items within the specific scales as shown in Table 4 should be regarded as tentative estimates only. Given our experience, the changes are expected to improve the parameter estimates.

## Discussion

We sought to conceptualise, develop and test a new measure of health literacy using modern and classical approaches to instrument development. We generated nine scales derived from the views of the general population, patients, healthcare professionals and policymakers. The items representing the constructs were carefully developed and tested in target populations and this indicated that nine distinct constructs were conceptually robust and that the items designed to measure them had good to excellent psychometric properties. We followed a validity-driven approach [29] involving numerous interviews, workshops, application in a calibration sample (N=634), application in a replication sample (n=405), and with constant attention to maximising measurement validity. The measure is now ready for further testing and validation of the interpretations of each scale’s data in the intended application settings; that is, applications in specific demographic groups, within health promotion, public health and clinical interventions, and in population health surveys.

Traditional approaches to the development of measures of complex multi-dimensional phenomena include undertaking literature reviews, reviews of items and scales in previously developed measures, and undertaking qualitative interviews with the target population to define the constructs within a predefined theoretical model [46]. Modern approaches also include systematic grounded approaches, where prevailing theories are eschewed until later in the development process, and great care is taken to fully understand the experiences and lives of stakeholders’ to develop constructs to serve these stakeholders [29]. We used the latter approach and consequently developed nine scales, some with constructs never before operationalised.

The scales cover a broad range of issues pertinent to an individual’s life and can be interpreted as intrinsic and extrinsic dimensions of health literacy. Some scales more strongly reflect: a) the capability of an individual to understand, engage with, and use health information and health services; or b) more strongly reflect the capability of an organisation to provide services that enable a person to understand, engage with and use their health information or services. The latter is based on the users’ lived experience of using health services. Consequently, we expect that the data from some scales will more strongly guide decisions about needs and outcomes at the individual level (Appraisal of health information, Social support, Actively managing my health), or at the organisational level (Feeling understood and supported by healthcare providers, Having sufficient information to manage my health), or provide guidance regarding both individual and organisational needs and outcomes (Ability to actively engage with healthcare providers, Navigating the healthcare system, Understanding health information well enough to know what to do). Clearly, responses to identified health literacy needs will involve a combined effort of interventions for individuals as well as organisational activities.

Even within one patient group, organisation or population, the variation of individual competencies across the nine areas is likely to be broad and all nine scales will generally need to be administered to provide a complete profile that captures the variety of health literacy needs. Most previous health literacy questionnaires have been tests of reading competencies in health-related contexts and were not intended to have good coverage of the currently available definitions of health literacy [19,47]. We used the definition of health literacy proposed by the WHO [1] as our starting point and as a touchstone against which we constantly re-assessed the adequacy of the emerging tool. The definition “…the cognitive and social skills which determine the motivation and ability of individuals to gain access to, understand and use information in ways which promote and maintain good health” was included in the development of the HLQ by revealing the lived experience of individuals and professionals through the concept mapping exercise. Given this inclusive starting point and our wide consultation, we expect that the HLQ will be a suitable tool in many Western and Eastern cultures, however it will be necessary to undertake rigorous studies to confirm its applicability in each setting [29].

The HLQ scales have strong to very strong psychometric properties and provide unique insights across nine separate areas. The robustness of the scales is attributed to two main activities: a) the efforts of generating cogent constructs grounded in peoples’ daily experiences, and b) efforts to generate and select high-quality items. An important innovation in questionnaire development was to write vignettes of people with high or low scores of this attribute. This assisted us to purposefully write items to cover the full breadth of the constructs in terms of degree of health literacy need or competency (item difficulty) and the range of types of needs or competencies within the construct.

The HLQ therefore will provide stakeholders (health and social care workers, managers and policymakers) with profiles of competencies or needs. When applied systematically, we expect that it will provide a useful reflection of an organisation’s needs or competencies to equitably serve its primary constituency. Many of the constructs are similar concepts to those that are often linked to the idea of empowerment. For example, Rappaport (1984) stated that "Empowerment is viewed as a process: the mechanism by which people, organizations, and communities gain mastery over their lives” [48]. Given that the HLQ dimensions provide detailed assessment of mechanisms by which a person can understand, access and use health information and health services, it may well come to be a useful operationalisation of empowerment in health.

Given that we took a validity-driven approach, it is important that we reflect on which draft constructs were present at the start of the process. Table 1 shows that there were initially 13 targets for measurement and 9 scales emerged in the final HLQ. It is critical that all of the initial elements are accounted for. We found that two constructs (Practicalities of accessing the health system and Beliefs and values) were not scalable because they were primarily a list of factors specific to contexts. We recommend that researchers compile these health literacy contextual factors as a set of individual questions. These may well be fundamental environmental and personal determinants of a person’s opportunity to access, understand and use information and healthcare. Some examples of contextual practical issues or beliefs include: the absence of public transport for citizens to travel to healthcare facilities; that vaccination for certain diseases is dangerous and should be avoided; that hospital care is the best first line care rather than primary care (i.e., family doctors).

One initial construct, Cognitive barriers/strategies, was subsumed into the Critical appraisal (later renamed Appraisal of health information) scale. We found that cognitive ability was part of a wide continuum of factors that ranged from being unable to consider health as a priority through to being able to make high level decisions about health (i.e., ability to appraise information). Finally, the Being health focused scale (later renamed Engagement in decisions) did not survive our validity-driven approach to scale development and is a gap in the HLQ that requires further work.

The HLQ captures previous notions of health literacy. The principle approach in North America has been to use health literacy as a link between literacy and a patient’s ability to safely comply with prescribed medication regimens [49]. This is well covered in the Institute of Medicine’s Prescription to End Confusion initiative [4] and the more recent National Action Plan to Improve Health Literacy [50,51]. The HLQ scale Understanding health information well enough to know what to do, will provide new patient-centred data on this link, as will the other eight scales. Each of the HLQ scales should provide new insight into how to improve health literacy, and critically, provide pertinent information for practitioners and healthcare organisations about which interventions might need to be put in place to optimise health outcomes.

Further evaluation of the HLQ indicates that it will also provide new opportunities to operationalise health literacy according to Nutbeam’s schema, a dominant approach in European and Asia-Pacific public health and health promotion circles [52]. Table 5 shows the definitions for Basic, Communicative and Critical Health Literacy Scales and how the HLQ might be used to capture the citizen’s capability at each of these levels. Our approach to item writing guided us to incorporate, where relevant, Bloom’s levels, and incidentally, Nutbeam’s levels, within and across scales. For example, one of the easiest scales is Understanding health information well enough to know what to do, with items such as ‘Follow the instructions from healthcare providers accurately’ (difficulty = 8%), and ‘Understand what healthcare providers are asking you to do’ (14%). These clearly relate to functional health literacy (Nutbeam’s 1^st^ level). The hardest item in this scale is ‘Read and understand all the information on medication labels’ (16%), which relates to more advanced cognitive and literacy skills, i.e., Nutbeam’s 2^nd^ level. The hardest HLQ scale is Appraisal of health information where the easiest item is ‘I compare health information from different sources’ (18%), which is harder than the hardest item on the Understanding health information scale and has elements of Nutbeam’s 2^nd^ level. The hardest item in the Appraisal scale is ‘I ask healthcare providers about the quality of the health information I find’ (38%), which calls for substantial critical appraisal skills (Nutbeam’s 3^rd^ level) for a respondent to endorse the ‘agree’ response option.

**Table 5:** **Linkage between the Nutbeam**[52]**schema of health literacy and the Health Literacy Questionnaire (HLQ)**

**Nutbeam schema[**[52]**]**	Broad matching HLQ domains*
**i) Basic/functional health literacy:** sufficient basic skills in reading and writing to be able function effectively in everyday situations.	9. Understanding health information well enough to know what to do
	2. Having sufficient information to manage my health
	8. Ability to find good quality health information
**ii) Communicative/interactive health literacy:** more advanced cognitive and literacy skills which, together with social skills, can be used to actively participate in everyday activities, to extract information and derive meaning from different forms of communication, and to apply new information to changing circumstances.	1. Feeling understood and supported by healthcare providers
	3. Actively managing my health
	4. Social support for health
	6. Ability to actively engage with healthcare providers
	7. Navigating the health system
	8. Ability to find good quality health information
**iii) Critical literacy:** more advanced cognitive skills, which together with social skills, can be applied to critically analyse information, and to use this information to exert greater control over life events and situations.	5. Appraisal of health information
	3. Actively managing my health
	4. Social support for health

* Within each HLQ scale there are some elements of the three levels of Nutbeam’s schema so overlap is expected

While the Nutbeam scheme was theory driven, and the HLQ was grounded in citizens’ lived experience and is validity-driven, the way forward to advance the health literacy field is the generation of outcomes data across a range of real-world settings. The use of the tool across settings over time, and with a wide range of weak and strong interventions, will generate a web of information about the HLQ performance, and then in turn, the HLQ may come to provide benchmark data by which researchers and policymakers can judge the relative value or impact of interventions in their fields.

While the HLQ comprises nine scales with good to excellent properties across several psychometric and conceptual parameters, further work is warranted. While the replication dataset had a low response rate (13.7%), the administration of the questionnaire was passive (one letter posted to patients after they returned home from a visit at an emergency department) with no follow-up or reminders. The setting was a regional public hospital (45% of Australians do not have private health insurance and therefore use public hospital services), with a large proportion of immigrants and refugees. While 13.7% is a low response rate, given the hospital’s catchment, and the purpose of this phase of the questionnaire development process, the data provide a reasonable challenge to the psychometric structure of the questionnaire.

The Appraisal of health information scale has lower reliability than what we intended (0.77), but this is still reasonable in this setting as a research tool. As mentioned above, we made a minor modification to the weakest item in this scale, which we expect will improve the reliability. Three other items were also modified, two with some minor disordered thresholds. The modifications were minor and intended to improve their performance so the estimates shown in Table 4 may be stronger in future studies. A further gap is the absence of data on sensitivity to change and test-retest reliability. These parameters are being examined in the OPtimising HEalth LIterAcy (OPHELIA) program, a large study to develop and test intervention options for the Victorian Health Literacy Response Framework, across eight disparate organisations, and will be reported in due course.

## Conclusions

Using systematic grounded methods and a validity-driven approach, we developed the Health Literacy Questionnaire which comprises a panel of nine independent indicators of health literacy that reflect important elements from the perspective of the general population, practitioners and policymakers. The nine scales capture a wide range of the lived experiences of people attempting to engage in understanding, accessing and using health information and health services. Importantly, the scales also provide a reflection of the quality of health and social service provision. Consistent with the validity-driven approach, the tool is now ready for application in the field where the interpretation of the scale scores requires validation in specific settings.

Given the instrument development approach we took, we expect that the HLQ will be useful in population surveys, studies of interventions, exploration of the needs of citizens, and studies of the needs and capabilities of individuals.

## Consent

Written informed consent was obtained from all patients taking part in face-to-face interviews. For patients completing postal surveys, the provision of patient information forms, statements that participation was voluntary, and the voluntary completion and return of the survey by patients constituted implied consent.

## Abbreviations

CFA: Confirmatory factor analysis; CFI: Comparative Fit Index; ESEM: Exploratory structural equation modeling; HLQ: Health Literacy Questionnaire; IRT: Item Response Theory; MI: Modification indices; OMERACT: Outcomes Measurement in Rheumatology; RMSEA: Root Mean Square Error of Approximation; SEPC: Standardised Expected Parameter Change; TLI: Tucker-Lewis Index; WSLMV: Weighted least squares mean and variance adjusted estimator; WRMR: Weighted Root Mean Square Residual.

## Competing interests

The authors declare that they have no competing interests.

## Authors’ contributions

The overall study design was led by RHO and RB. RWB led the validity-driven approach and construct conceptualisation. GRE led the quantitative analysis. RHO, RB, RWB, GRE and MH wrote the items. All authors contributed to data collection and interpretation. RHO, RB and RWB wrote the initial draft and all authors contributed to redrafting and approving the final draft.

## Pre-publication history

The pre-publication history for this paper can be accessed here:

http://www.biomedcentral.com/1471-2458/13/658/prepub
